# Monitoring persistence of the entomopathogenic fungus *Metarhizium anisopliae* under simulated field conditions with the aim of controlling adult *Aedes aegypti* (Diptera: Culicidae)

**DOI:** 10.1186/1756-3305-7-198

**Published:** 2014-04-25

**Authors:** Aline T Carolino, Adriano R Paula, Carlos P Silva, Tariq M Butt, Richard I Samuels

**Affiliations:** 1 Department of Entomology and Plant Pathology, Universidade Estadual do Norte Fluminense Darcy Ribeiro, Campos dos Goytacazes, Rio de Janeiro 28013-602, Brazil; 2 Departamento de Bioquímica, Universidade Federal de Santa Catarina, Florianópolis, Santa Catarina 88040-900, Brazil; 3 Department of Biosciences, Swansea University, Swansea, UK

**Keywords:** Persistence, Viability, Vector, Dengue, Oil formulation

## Abstract

**Background:**

Entomopathogenic fungi are potential candidates for use in integrated vector management, with recent emphasis aimed at developing adult mosquito control methods. Here we investigated the persistence of the fungus *Metarhizium anisopliae* when tested against female *A. aegypti* under field conditions.

**Methods:**

Black cotton cloths impregnated with *M. anisopliae* conidia, formulated in vegetable oil + isoparaffin, were maintained on a covered veranda for up to 30 days. At specific times, pieces of the cloths were removed, placed in Tween 80 and the resuspended conidia were sprayed directly onto mosquitoes. The persistence of conidia impregnated on black cloths using three different carriers was evaluated in test rooms. Fifty mosquitoes were released into each room and after a 5 day period, the surviving insects were captured. Another 50 insects were then released into each room. The capacity of the fungus at reducing mosquito survival was evaluated over a total of 35 days.

**Results:**

Conidia extracted from cloths maintained on the veranda for 2 to 18 days remained virulent, with 28 to 60% mosquito survival observed. Mosquito survival following exposure to fungus impregnated cloths showed that fungus + Tween caused similar reductions to that of fungus + vegetable oil. Mosquitoes exposed to the formulation fungus + vegetable oil had survival rates of 36% over the first 5 days of the experiment. Following the release of the second cohort of mosquitoes (6-11days), survival increased to 50%. The survival of the 12–17 day cohort (78%) was statistically equal to that of the controls (84%). Formulation of the fungus in vegetable oil + isoparaffin increased the persistence of the fungus, with the 18–23 day cohort (64% survival) still showing statistical differences to that of the controls (87% survival).

**Conclusions:**

The potential of entomopathogenic fungi for the control of adult *A. aegypti* was confirmed under field conditions. Vegetable oil + isoparaffin formulations of *M. anisopliae* significantly increased the effectiveness of the fungus, thus reducing the need for frequent changes of black cloths in residences. Our future aim is to obtain effective control of mosquito populations, with cloths only needing to being replaced once a month.

## Background

The use of entomopathogenic fungi against insect disease vectors such as malaria mosquitoes is currently the subject of extensive research since the publication of two key papers in 2005 [1,2]. There seems little doubt that entomopathogenic fungi will play an important role in reducing the suffering caused by vector borne diseases, however, field testing and strategies for application of fungi require further research. Recent studies have shown the effectiveness of odor baited stations impregnated with fungus (*Metarhizium anisopliae*) for the control of *Anopheles arabiensis*[3]. Point source spray application of *M. anisopliae* to the inside of clay water storage pots was shown to be efficient at reducing *Anopheles gambiae s.s.* and *An. funestus* survival under laboratory conditions [4]. The use of *Beauveria bassiana* impregnated mesh screens has been tested against *A. gambiae s.s.* under laboratory conditions [5]. A range of materials was tested with the potential for use as eave curtains in African dwellings. The first field test of a fungal pathogen for the control of malaria mosquitoes was carried out in Africa, by suspending large cotton sheets impregnated with fungi to the ceiling of dwellings [2]. These field trials were promising, resulting in a 23% infection rate, which when plugged into a model of malaria transmission to derive an entomological inoculation rate, predicted a 75% reduction in malaria transmission. Recently an intra-domicile method was tested using fungus treated baffles attached to the eaves of houses or fungus-treated strips of cloth placed around an occupied bed net. These methods resulted in a 39-57% reduction in mosquito survival [6].

Cage trials (115 × 60 × 75 cm) were carried out to test the effectiveness of *M. anisopliae* impregnated black cotton cloths (30 × 20 cm) for the control of the dengue vector *Aedes aegypti*[7]. These results showed a 70% reduction in survival over a 7 day exposure period to the fungus. In Australia, the effect of *B. bassiana* on survival, blood-feeding behaviour and fecundity was tested against *A. aegypti* in large cages (5 × 7 × 4 m), with results showing an 80% reduction in blood-feeding and a reduction in mosquito survival of 59 - 95% [8]. However, in these experiments the mosquitoes were infected under laboratory conditions and then released into the cages to observe modifications in behavior and survival. Paula and co-workers [9] demonstrated the relationship between the length of time mosquitoes were exposed to fungus impregnated black cloths and survival under field conditions in large cages (115 × 60 × 75 cm). Significant reductions in survival were observed when black cloths were left in the cages for 12 h. Longer exposure times resulted in further reductions in survival. A recent study by our group was the first to demonstrate the efficiency of fungus impregnated black cotton cloths at reducing *A. aegypti* survival when tested in rooms simulating intra-domicile conditions [10]. Following 7 days, the survival rate of fungus exposed insects was 44%, a significant reduction when compared to control survival (76%). The use of the neonicotinoid insecticide imidacloprid together with the fungus further reduced survival rates to 38% [10].

Because these entomopathogens are sensitive to environmental conditions, oil-based formulations show potential for promoting protection of conidia and facilitating adhesion onto hydrophobic surfaces of the insect integument [11]. Furthermore, oil formulations have been shown to increase the tolerance of conidia to abiotic factors [12,13].

Formulation of entomopathogenic fungi in the synthetic oil Shellsol® (an isoparaffin based product) has been used with the aim of increasing persistence and virulence of fungi [14]. The experiments performed by Bukhari and co-workers [15] showed an increased persistence of isolates of *M. anisopliae* and *B. bassiana* formulated in oil for use against larvae of *Anopheles*. In experiments with adults of the same vector species, Mnyone and co-workers [16] observed in laboratory conditions, the persistence of both *M. anisopliae* and *B. bassiana* when impregnated on white netting, mud panels or black cloths exposed to *Anopheles* mosquitoes. In that study the fungi were formulated in a 1:1 mixture of the highly refined oils Enerpar® and Shellsol®. Fungus impregnated black cotton cloths and mud panels were more effective at reducing mosquito survival than white polyester netting, with significant reductions in survival observed for up to 28 days.

The current study evaluated the persistence of the fungus *M. anisopliae* formulated in three different carriers and applied to black cotton cloths with the aim of controlling *A. aegypti* adults under extra- and intra-domicile field conditions.

## Methods

### Maintenance of insect colonies

*A. aegypti* (Rockefeller strain) colonies were reared in cages at 25°C; approximately 75% RH; 16:8 L/D photoperiod and provided with a 10% sucrose solution. Insects were provided with blood meals by placing a mouse, immobilized in a wire mesh bag, in the adult mosquito cages. Following the blood meal, oviposition occurred in beakers half filled with water and lined with filter paper placed in adult cages. Larval eclosion was stimulated by total immersion of the filter paper in water to which mouse food had been previously added (24 h) to reduce oxygen levels.

Larvae were maintained in plastic trays (80 larvae per 100 mL) and fed on freshly ground commercial mouse food (0.05 g per L) until reaching the pupal stage. Pupae were separated into water filled beakers and transferred to cages before adult emergence. Recently hatched (2–3 days old) females were used for all experiments.

### Fungal isolate and preparation of suspensions

The isolate of *M. anisopliae* used here was obtained from the collection at ESALQ (ESALQ818) in Piracicaba (São Paulo), which had been previously demonstrated to have high virulence against adult *A. aegypti*[7]. Fungi were cultured on Sabouraud Dextrose Agar (Dextrose 10 g; Peptone 2.5 g; Yeast Extract 2.5 g; Agar 20 g in 1 L H_2_0) at 27°C for 15 days before being used in experiments. Fungal suspensions were initially prepared in Tween 80 (0.05% in sterile distilled water) and conidial concentration determined using a Neubauer hemocytometer. A final concentration of 1×10^9^ conidia mL^-1^ was prepared by serial dilution.

### Persistence of fungal virulence under extra-domicile conditions

The virulence of *M. anisopliae* (ESALQ 818) to female *A. aegypti* when formulated in vegetable oil (sunflower oil; Sadia Ltd. Brazil) + synthetic oil (isoparaffin; BroadLub Ltd. Brazil) and maintained in natural conditions was evaluated over time. Black cotton cloths (20 x 10 cm) were first sterilized and then immersed in conidial suspensions (1×10^9^ conidia mL^-1^). The cloths were then left to dry for 16 h in a controlled temperature room (26°C, 71 ± 7% RH and 12 h L:D cycle). The final concentration of conidia on the cloths was approximately 1×10^8^ conidia cm^2^, which was estimated by removing 1 cm^2^ samples of the cloth and resuspending the conidia in Tween. The conidial concentration was then determined using a hemocytometer. Black cloths were then hung on a clothes line and maintained on a covered veranda (max 32.6°C, min 21.5°C; max 74.5%, min 45.3% RH), without exposure to direct sunlight, for 2, 6, 12, 18, 24, and 30 days. Following each time period, 1 cm^2^ pieces of the cloths were removed and placed in 1 mL Tween 80 (0.05%) and vigorously vortex mixed. The conidial suspension was then sprayed directly onto CO_2_ anesthetized 2–3 day old females using a Potter tower (Burkhart Ltd. UK). Following spraying, the insects were placed in plastic pots (10 per pot) covered with netting (12 cm diameter × 7 cm high). All groups were offered filter paper discs soaked in 10% sucrose placed on the netting surface of the pots, which were changed on a daily basis. Survival of insects was determined daily for a 7 day period and dead insects were removed during observations. All experiments were carried out three times with a minimum of 30 insects per treatment or control group. The homogeneity of the replicate experiments was determined using the Log-rank test at the 95% significance level and subsequently the results were pooled for survival curve analysis (Gehan-Breslow-Wilcoxon test). Analysis was performed using GraphPad software. Confirmation of mortality as a result of fungal infection was not carried out due to the high levels of saprophytic colonization of the cadavers.

### Persistence of fungal virulence in simulated dwellings

Two identical rooms (3 × 2 × 2.3 m) each with a window, 2 chairs and 2 desks were used for these experiments. The rooms were first prepared for experiments by cleaning with disinfectant and three wick feeders placed in each room with 30 ml 10% sucrose. Black cotton cloths (20 × 10 cm) were prepared as previously stated above. Cloths were immersed in suspensions of ESALQ818 formulated in Tween 80 (0.05%), vegetable oil or vegetable oil + isoparaffin. Control treatment cloths were immersed in Tween 80 (0.05%), vegetable oil or vegetable oil + isoparaffin without fungus. For each repetition, one of the rooms was chosen randomly for application of cloths with fungi whilst the other room was used for control treatments. Therefore, one of the rooms contained cloths treated with fungus whilst the other room contained cloths treated with carrier only.

For each experimental room, 5 cloths were fixed under furniture using “silver tape” (3 M Ltd, Brazil). Fifty female *A. aegypti* were then released into each room and the doors sealed with masking tape (see Additional file 1 for photograph of test room). At the end of the fifth day of the experiment, a trap (BG-Sentinel™ Biogents Ltd. Germany) was quickly placed in the room and 24 h later the number of captured mosquitoes recorded. The room was then inspected for any mosquitoes that had failed to be captured by the BG-Sentinel™, in which case mosquitoes were captured using a suction trap before releasing the next cohort. This procedure was repeated five times during the period of the experiment, thus the efficiency of the fungus at reducing mosquito survival was evaluated over the following periods: 0–5, 6–11, 12–17, 18–23, 24–29 and 30–35 days (see Additional file 2 for flow diagram of experimental procedure). All experiments were carried out three times. Survival rates were compared using one-way analysis of variance and Tukey’s test (SPSS 14 software). A Data-logger (Watch-Dog, USA) was used to monitor temperature and humidity hourly during all experiments (temperature max: 28.8°C min: 25.7°C; RH max: 78.4% min: 61.7%).

### Ethical approval

The use of live animals to feed female mosquitoes was approved by the UENF Ethical Committee.

## Results

The formulation of ESALQ 818 in oil vegetable oil, isoparaffin or a mixture of both oils had no negative effect on conidial germination as seen by suspending conidia in oil and then carrying out plate count germination tests. All treatments resulted in >80% germination when monitored over a 20 day period (results not shown). However, viability was reduced to zero when conidia were maintained in Tween 80 (0.05%) for two days at room temperature.

**Figure 1:**
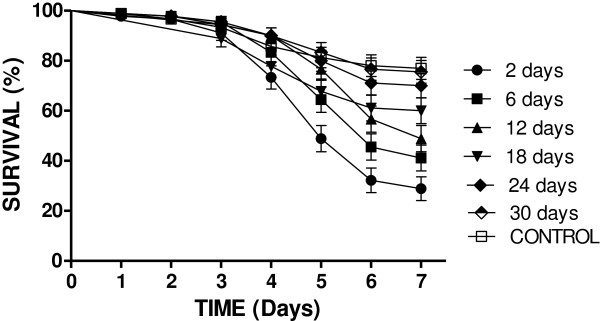
**Survival curves of female *Aedes aegypti *sprayed with conidia resuspended from black cloths that had been exposed to extra-domicile conditions for up to 30 days.** Note: The control survival curve is the mean of all the control groups (6) used for each time point. Results are the means of three experiments with 30 insects used per experiment.

In the first field trial, conidia were formulated in vegetable oil + isoparaffin and exposed to natural extra-domicile conditions for different lengths of time before performing survival bioassays (Figure 1 and Table 1). Figure 1 shows the survival curves for each time period that the cloths had been maintained on the veranda. The lowest survival rates (28%) were seen when mosquitoes were sprayed with conidia that had been exposed to extra-domicile conditions for 2 days only. Following a 12 day exposure to ambient conditions, the fungus only reduced survival to ~50% over the bioassay period. The survival curves demonstrate the steady decline in virulence of the conidia over time.

**Table 1:** Percentage survival of mosquitoes following spray application of conidia retrieved from cloths that had been left under natural extra-domicile conditions for periods of 2 to 30 days

**Period exposed to natural conditions (days)**	**% end point survival**	**χ**^**2**^**(df = 1)**	***p***
2	28.8	31.01	*<*0.0001*
6	41.1	16.5	*<*0.0001*
12	48.8	9.01	0.0027*
18	60	5.26	0.0218*
24	70	0.578	0.4471
30	70	0.0004	0.9894
CONTROL^$^	76.6	-	-

Gehan-Breslow-Wilcoxon test for pair-wise survival curve comparisons between the period the black cloths were exposed to natural conditions and control survival.

*significantly different to control survival at the 5% level.

^$^The result for the controls was the pooled value for control insects at each of the six time points and three repetitions.

Table 1 shows pair-wise survival curve comparisons for each time period (when compared to their respective controls) and demonstrated that conidia from cloths maintained on the veranda for 2 to 18 days were “virulent”, causing reductions in survival of 28 to 60%, which was statistically different from that of the controls (76% survival). Following 24 days of exposure to natural conditions, the conidia no longer caused any significant negative effects on mosquito survival, in other words, their was no statistical difference in survival from that of the control treatment (χ^2 ^0.578; *p* = 0.44).

In an alternative experiment to test conidial virulence over time, insect survival when *A. aegypti* females were exposed to fungus impregnated cloths fixed under furniture in two test rooms was evaluated over a 35 day period (Table 2).

**Table 2:** **Mean survival rates of *Aedes aegypti *** **released into rooms containing black cloths impregnated with fungal conidia using three different carriers**

**Survival (%)**			
**Time since cloths first placed in rooms (days)**	**Conidia + T**	**Conidia + V**	**Conidia + V + I**
0–5	38 ± 1 b	36.6 ± 1.5 b	32.6 ± 2.08 d
6-11	49.3 ± 2.51 b	50 ± 2.64 b	40.6 ± 0.57d
12 -17	81 ± 2.08 a	78.6 ± 2.80 a	60 ± 2.64 c
18-23	82 ± 1 a	81.3 ± 3.21 a	64.6 ± 2.51 bc
24-29	ND	82.6 ± 1.52 a	77.3 ± 0.57 ab
30-35	ND	ND	83.3 ± 2.51 a
**Control**	83.3 ± 2.51 a	84.6 ± 2.08 a	87.3 ± 4.16 a

Three fungal treatments were tested: conidia + Tween (T), conidia + vegetable oil (V) and conidia + vegetable oil + isoparaffin (I).

At the end of each time period, the surviving mosquitoes were captured and a new cohort released into the room. Mean survival values followed by the same letter indicate that the results for each treatment were not statistically different when using a one way analysis of variance followed by Tukey’s test (5% probability) to compare the different periods that cloths had been left in the rooms. ND = not determined. Control results for each time period were not significantly different for each repetition (P > 0.01) and were thus pooled and shown as a single mean value for each treatment.

In the first part of this experiment, female *A. aegypti* survival rates when exposed to black cloths impregnated with conidia suspended in Tween 80 were monitored. A one way ANOVA showed differences between time periods (F_(4, 14)_ = 92.46; *p* < 0.01). The survival rates during the periods 0–5 days and 6–11 days were significantly different to the controls, however, the survival rates of all groups from day 12 onwards were equal to that of the controls (*p* > 0.05).

A one way ANOVA showed differences between time periods for the experiment using cloths impregnated with conidia + vegetable oil (F_(5, 17)_ = 58.83; *p* < 0.01). Exposure of mosquitoes to cloths impregnated with Conidia + vegetable oil resulted in a survival rate of 36% over the first 5 days of the experiment (Table 2). Following the release of the second group of mosquitoes (6–11 days), survival increased to 50%. The survival of the 12–17 day group (78%) was statistically equal to that of the controls (84%; *p* > 0.05). The ANOVA for the experiment using the formulation Conidia + V + I showed that there were statistical differences between time periods (F_(6, 20)_ = 54.96; *p* < 0.01). Formulation of the fungus in vegetable oil + isoparaffin increased persistence, with the 18–23 day group (64% survival) statistically different to that of the controls (87% survival). The survival rate of the next test cohort, 24–29 days (70% survival), showed no statistical difference to the controls (*p* > 0.05).

## Discussion

Although entomopathogenic fungi are being extensively researched as biocontrol agents for the adult stage of disease vectoring mosquitoes, few studies have been carried out under field conditions on the infection of the dengue vector *A. aegypti*. One of the first studies, simulating field conditions, was carried out recently using black cotton cloths impregnated with conidia of *Metarhizium anisopliae*[10], which showed the efficiency of this method at reducing mosquito survival over a 7 day period. In the current study, the efficiency of fungus impregnated black cloths maintained in ambient conditions in reducing mosquito survival over longer time periods was tested. Two types of mosquito survival bioassays were performed; (1) verifying the virulence over time of conidia that had been maintained under extra-domicile conditions and (2) successively releasing cohorts of female mosquitoes into rooms with fungus impregnated cloths attached to furniture. In the first bioassay, conidia that had been maintained under extra-domicile conditions were extracted from the cloths and sprayed directly onto mosquitoes. This experiment showed the steady and rapid decline in fungal virulence over time with initial survival rates of 28% (day 2) increasing to approximately 50% when using conidia that had been exposed to extra-domicile conditions for 12 days. Similar results were obtained for *A. gambiae* exposed to *M. anisopliae* and *B. bassiana* formulated in oil over a 28 day period, although all experiments were carried out under controlled laboratory conditions (26-27°C; RH 85-95%), thus it is unknown if fluctuating environmental conditions would affect residual activity [17].

Previous studies have demonstrated that biotic and abiotic factors can negatively effect fungal germination and hyphal growth [18]. Unfavorable temperatures, humidity and ultra-violet radiation can also rapidly affect conidial viability. Thermo-tolerance of *M. anisopliae* has been correlated to geographical origin of isolates [19], indicating that isolates obtained from the area where the fungus is planned to be used would be more tolerant of ambient conditions normally found in that region.

The main strategy here is to use black cotton cloths impregnated with *M. anisopliae* for the control of adult *A. aegypti*, by placing these cloths in strategic extra- and intra-domicile locations. These locations will be chosen to preferably not receive direct sun-light as this would be damaging to the conidia. Conidial inactivation caused by UV radiation is one of the factors responsible for reducing the efficiency of fungi in biological control programs. Exposure to both UVA and UVB rapidly reduced fungal viability as documented in studies on the effect of radiation on conidial germination [20,21]. With the aim of increasing conidial persistence in the presence of UV, Inglis and co-workers [22] tested the protective effects of oil and sunscreens on *B. bassiana*. Conidial suspensions in parafinic oil decreased conidial mortality rates from 97% (water suspensions) to 74% during a 1 h exposure to UV. The successful use of *Metarhizium* for the control of the desert locust *Schistocera gregaria* in Africa can be attributed to the formulation of conidia in oil, increasing the persistence in the field and reducing the dependence on high humidity conditions for host infection [23]. The oil formulations also facilitate application using ultra-low volume spraying equipment [23]. Different oil formulations were tested for UV protective properties by Alves and co-workers [12]. Peanut oil and Shellsol plus Ondina protected *M. anisopliae* conidia against the deleterious effects of a 6 h exposure to UV light significantly better than the other formulations tested. Emulsifiable oil fungal formulations such as water plus Emoleo®, water plus Codacide®, water plus Ashlade® and water plus Natur’l oil ® also provided significantly improved protection of conidia against UV light compared with the conventional water plus 0.05% Tween 80 formulation.

The results for mosquito survival when testing for conidial persistence in rooms simulating human residences, comparing conidial suspensions in three different carriers used to impregnate the cloths, showed no difference between Tween 80 and vegetable oil. However, conidial suspensions in Tween maintained at approximately 25°C for 48 h did not germinate. Therefore, Tween could not be used to store conidia. Conidial suspensions in the two oil carriers remained viable for more than 30 days at 25°C (>80% germination). These results demonstrate the marked difference between germination bioassays and virulence bioassays. Whilst conidial suspensions in Tween applied to cloths continued to kill mosquitoes under field conditions for up to 11 days, this carrier was unsuitable for fungal storage. The use of a mixture of vegetable oil and isoparaffin, not only maintained conidial viability of stored propagules, but also increased the length of time that conidia were remained virulent under field conditions.

*B. bassiana* conidial suspensions in Shellsol T were applied to polyester netting which was subsequently exposed to natural environmental conditions in West Africa, and then conidial viability (germination) was determined over a 20 day period [14]. The results showed a steady decline in viability when fungus impregnated netting was stored at 24.9 - 38.6°C and a humidity ranging from 70 - >95% RH, however, viability remained high and stable over time when conidial suspensions in oil were maintained refrigerated. Scholte and co-workers [2] found that fungal viability was reduced from 96% to 63% after three weeks exposure to field conditions in Tanzania.

Previous studies have shown that a prolonged period (≥2 days) of elevated humidity (≥96%) was a pre-requisite for high fungal infection rates (>50%) of *Rhodnius prolixus* by *B. bassiana*[24]. However as seen here, at no time during the experiments in the simulated dwelling trials, did relative humidity exceeded 80%. Even so, exposure of mosquitoes to fungus impregnated cloths still significantly reduced survival rates. Significant reductions in female *A. aegypti* survival rates were also obtained under apparently non-optimum environmental conditions (temperature max: 28.8°C min: 25.7°C; RH max: 78.4% min: 61.7%) when releasing mosquitoes into rooms simulating a human residence together with fungus impregnated black cloths [10].

One interesting advantage of using entomopathogenic fungi for the control of adult mosquitoes is that fungi could be applied to surfaces not directly exposed to sunlight such as bed-nets, intra-domicile walls, mosquito screens and other surfaces such as strategically placed cloths. In the case of the studies carried out by our group, the strategic positioning of fungus impregnated cloths in locations not subject to direct sunlight would benefit the persistence of the fungus in the field. It is interesting to note that adult *A. aegypti* normally rest on dark surfaces or in dark locations. Black surfaces are known to attract a range of mosquito species [25], although other studies have shown that stationary objects of low reflectance and solid color were the most attractive to male and female *A. aegypti*[26]. A recent study by our group has shown the landing frequencies of female *A. aegypti* on black cotton cloths during natural photoperiods [10]. Mosquitoes were attracted to the cloths (highest numbers of mosquitoes present on the cloths) during daylight hours, indicating that this behavior could be related to predator avoidance as the insects are very difficult to see when resting on the cloths. These experiments also showed that insects were not repelled by the presence of conidia on the cloths.

The results seen here when using a mixture of vegetable and isoparaffin oil carriers, show significant advantages when compared to either vegetable oil or Tween alone. However, the aim of maintaining high levels of conidial viability under field conditions for up to one month was not achieved and further studies are now being carried out to improve persistence.

## Conclusions

The potential of the entomopathogenic fungus *M. ansiopliae* for the control of adult *A. aegypti* was confirmed here under field conditions. The use of a mixture vegetable and synthetic oil as a carrier for the conidia when applied to black cotton cloths significantly increased fungal persistence and thus maintained virulence over extended time periods in intra and extra-domicile conditions, reducing survival of mosquitoes that came into contact with the cloths. Further work on formulating the conidia will be aimed at maintaining high levels of virulence for at least a one month period.

## Competing interests

The authors declare that they have no competing interests.

## Authors’ contributions

ATC carried out the experiments and participated in the design of the study. ARP performed the statistical analysis, helped carry out experiments and maintained the insect colonies. CPS and TMB participated in the design of experiments and writing of the manuscript. RIS conceived the study, participated in its design, supervised the experiments and wrote the manuscript. All authors read and approved the final manuscript.

## Supplementary Material

Additional file 1 Room used for testing fungus impregnated black cloths. Click here for file

Additional file 2 **Flow diagram of experimental procedure used for testing conidial persistence under inter-domicile conditions.** Note that the flow diagram is representative of the experiment until day 11 only. Click here for file

## References

[B1] Blanford S Chan BHK Jenkins N Sim D Turner RJ Read AF Thomas MB Fungal pathogen reduces potential for malaria transmission Science 2005 308 1638 1641 10.1126/science.1108423 15947189

[B2] Scholte EJ Ng’habi K Kihonda J Takken W Paaijmans K Abdula S Killeen GF Knols BGJ An entomopathogenic fungus for control of adult African malaria mosquitoes Science 2005 308 1641 1642 doi:10.1126/science.1108639 10.1126/science.1108639 15947190

[B3] Lwetoijera DW Sumaye RD Madumla EP Kavishe DR Mnyone LL Russell TL Okumu FO An extra-domiciliary method of delivering entomopathogenic fungus, *Metarhizium anisopliae* IP 46 for controlling adult populations of the malaria vector, *Anopheles arabiensis* Parasit Vectors 2010 3 18 10.1186/1756-3305-3-18 20233423 PMC2848008

[B4] Farenhorst M Farina D Scholte EJ Takken W Hunt RH Coetzee M Knols BGJ African water storage pots for the delivery of the entomopathogenic fungus *Metarhizium anisopliae* to the African malaria vectors *Anopheles gambiae s.s.* and *An. funestus* Am J Trop Med Hyg 2008 78 910 916 18541768

[B5] Farenhorst M Hilhorst A Thoma MB Knols BGJ Development of fungal applications on netting substrates for malaria vector control J Med Entomol 2011 48 2 305 313 doi:10.1603/ME10134 10.1603/ME10134 21485366

[B6] Mnyone LL Lyimo IN Lwetoijera DW Mpingwa MW Nchimbi N Hancock PA Russell TL Kirby MJ Takken W Koenraadt CJM Exploiting the behaviour of wild malaria vectors to achieve high infection with fungal biocontrol agents Malar J 2012 11 87 10.1186/1475-2875-11-87 22449130 PMC3337815

[B7] Paula AR Brito ES Pereira CR Carrera MP Samuels RI Susceptibility of adult *Aedes aegypti* (Diptera: Culicidae) to infection by *Metarhizium anisopliae* and *Beauveria bassiana*: prospects for Dengue vector control Biocont Sci Tech 2008 18 1017 1025 10.1080/09583150802509199

[B8] Darbro JM Johnson PH Thomas MB Ritchie SA Kay BH Ryan PA Effects of *Beauveria bassiana* on Survival, Blood-Feeding Success, and Fecundity of *Aedes aegypti* in Laboratory and Semi-Field Conditions Am J Trop Med Hyg 2012 86 656 664 10.4269/ajtmh.2012.11-0455 22492151 PMC3403760

[B9] Paula AR Carolino AT Silva CP Samuels RI Efficiency of fungus-impregnated black cloths combined with Imidacloprid for the control of adult *Aedes aegypti* (Diptera: Culicidae) Lett Appl Microbiol 2013 57 157 163 doi:10.1111/lam.12090 10.1111/lam.12090 23607802

[B10] Paula AR Carolino AT Silva CP Pereira CR Samuels RI **Testing fungus impregnated cloths for the control of adult**** *Aedes aegypti* ****under natural conditions.** Parasit Vectors 2013 6 256 10.1186/1756-3305-6-256 24010874 PMC3848359

[B11] Luz C Batagin I Potential of oil-based formulations of *Beauveria bassiana* to control *Triatoma infestans* Mycopathologia 2005 160 51 62 10.1007/s11046-005-0210-3 16160769

[B12] Alves RT Bateman RP Gunn J Prior C Leather SR Effects of Different Formulations on Viability and Medium-Term Storage of *Metarhizium anisopliae* Conidia Neotrop Entomol 2002 31 91 99 10.1590/S1519-566X2002000100013

[B13] Leucona RE Edelstein JD Berretta MF Rossa FRL Arcas JA Evaluation of *Beauveria bassiana* (Hyphomycetes) Strains as Potential Agents for Control of *Triatoma infestans* (Hemiptera: Reduviidae) J Med Entomol 2001 38 172 179 10.1603/0022-2585-38.2.172 11296819

[B14] Howard AFV N’Guessan R Koenraadt CJM Asidi A Farenhorst M Akogbéto M Knols BGJ Takken W First report of the infection of insecticide-resistant malaria vector mosquitoes with an entomopathogenic fungus under field conditions Malar J 2011 10 24 doi:10.1186/1475-2875-10-24 10.1186/1475-2875-10-24 21288359 PMC3045381

[B15] Bukhari T Takken W Koenraadt CJM Development of *Metarhizium anisopliae* and *Beauveria bassiana* formulations for control of malaria mosquito larvae Parasit Vectors 2011 4 23 10.1186/1756-3305-4-23 21342492 PMC3051916

[B16] Mnyone LL Kirby MJ Lwetoijera DW Mpingwa MW Simfukwe ET Knols BGJ Takken W Russell TL Tools for delivering entomopathogenic fungi to malaria mosquitoes: effects of delivery surfaces on fungal efficacy and persistence Malar J 2010 9 246 10.1186/1475-2875-9-246 20799967 PMC2939623

[B17] Mnyone LL Kirby MJ Lwetoijera DW Mpingwa MW Knols BGJ Takken W Russell TL Infection of the malaria mosquito, *Anopheles gambiae*, with two species of entomopathogenic fungi: effects of concentration, co-formulation, exposure time and persistence Malar J 2009 8 309 doi:10.1186/1475-2875-8-309 10.1186/1475-2875-8-309 20030834 PMC2808315

[B18] Roy HE Steinkraus DC Eilenberg J Hajek AE Pell JK Bizarre Interactions and Endgames: Entomopathogenic Fungi and their Arthropod Hosts Annu Rev Entomol 2006 51 331 357 doi:10.1146/annurev.ento.51.110104.150941 10.1146/annurev.ento.51.110104.150941 16332215

[B19] Rangel DEN Braga GUL Anderson AJ Roberts DW Variability in conidial thermotolerance of *Metarhizium anisopliae* isolates from different geographic origins J Invertebr Pathol 2005 88 116 125 10.1016/j.jip.2004.11.007 15766928

[B20] Braga GUL Flint SD Miller CD Anderson AJ Roberts DW Variability in response to UV-B among species and strains of *Metarhizium* isolated from sites at latitudes from 61°N to 54°S J Invertebr Pathol 2001 78 98 108 10.1006/jipa.2001.5048 11812112

[B21] Braga GUL Flint SD Miller CD Anderson AJ Roberts DW Both solar UVA and UVB radiation impair conidial culturability and delay germination in the entomopathogenic fungus *Metarhizium anisopliae* Photochem Photobiol 2001 74 734 739 10.1562/0031-8655(2001)074<0734:BSUAUR>2.0.CO;2 11723803

[B22] Inglis GD Goettel MS Johnson DL Influence of ultraviolet-light protectants on persistence of the entomopathogenic fungus, *Beauveria bassiana* Biol Cont 1995 5 581 590 10.1006/bcon.1995.1069

[B23] Lomer CJ Bateman RP Johnson DL Langewald J Thomas M Biological control of locusts and grasshoppers Annu Rev Entomol 2001 46 667 702 doi:10.1146/annurev.ento.46.1.667 10.1146/annurev.ento.46.1.667 11112183

[B24] Fargues J Luz C Effects of Fluctuating Moisture and Temperature Regimes on the Infection Potential of *Beauveria bassiana* for *Rhodnius prolixus* J Invertebr Pathol 2000 75 202 211 doi:10.1006/jipa.1999.4923 10.1006/jipa.1999.4923 10753596

[B25] Hecht O Hernandez-Corzo J On the visual orientation of mosquitoes in their search of resting places Entomol Exper et Appl 1963 6 63 74 doi:10.1111/j.1570-7458.1963.tb00603.x 10.1111/j.1570-7458.1963.tb00603.x

[B26] Muir LE Kay BH Thorne MJ *Aedes aegypti* (Diptera: Culicidae) vision: response to stimuli from the optical environment J Med Entomol 1992 29 445 450 1625292 10.1093/jmedent/29.3.445

