# Supplementary figures and images for: Monitoring persistence of the entomopathogenic fungus Metarhizium anisopliae under simulated field conditions with the aim of controlling adult Aedes aegypti (Diptera: Culicidae)
Source: Parasit Vectors. 2014 Apr 25;7:198. doi: 10.1186/1756-3305-7-198 (PMC4021620; doi:10.1186/1756-3305-7-198)

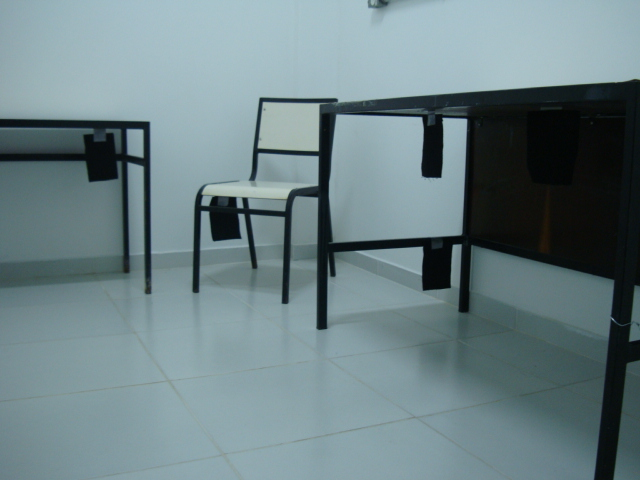


Room used for testing fungus impregnated black cloths.

Supplement: Additional file 1 — Room used for testing fungus impregnated black cloths. [file 1756-3305-7-198-S1.doc]
