# Supplementary material for: Monitoring persistence of the entomopathogenic fungus Metarhizium anisopliae under simulated field conditions with the aim of controlling adult Aedes aegypti (Diptera: Culicidae)
Source: Parasit Vectors. 2014 Apr 25;7:198. doi: 10.1186/1756-3305-7-198 (PMC4021620; doi:10.1186/1756-3305-7-198)
Supplement: Additional file 2 — Flow diagram of experimental procedure used for testing conidial persistence under inter-domicile conditions. Note that the flow diagram is representative of the experiment until day 11 only. [file 1756-3305-7-198-S2.ppt]

## Slide 1
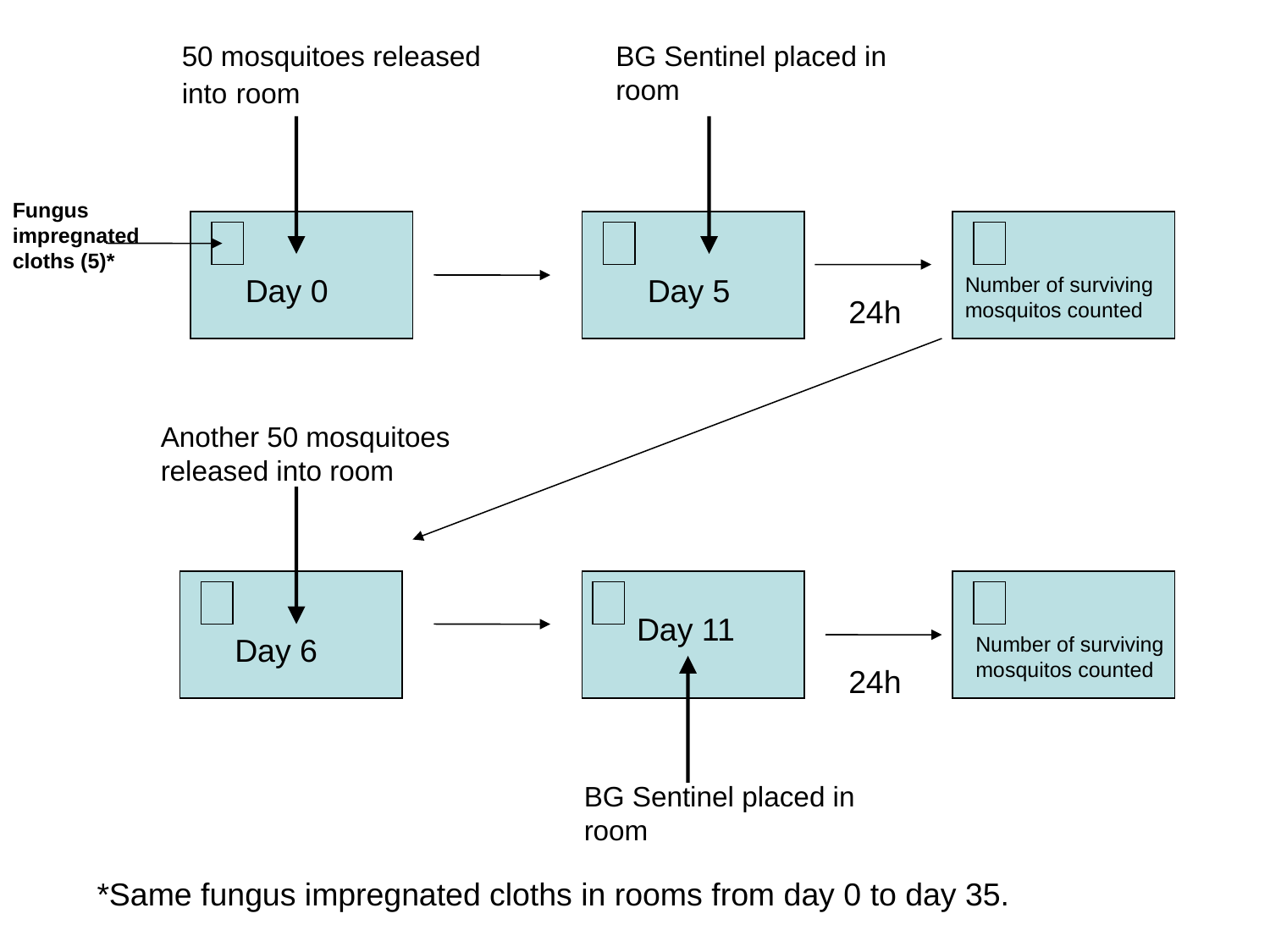

50 mosquitoes released into room
BG Sentinel placed in room
Fungus impregnated cloths (5)*
Day 0
Day 5
Number of surviving mosquitos counted
24h
Another 50 mosquitoes released into room
Day 11
Day 6
Number of surviving mosquitos counted
24h
BG Sentinel placed in room
*Same fungus impregnated cloths in rooms from day 0 to day 35.
